# Beyond the Coral Triangle: high genetic diversity and near panmixia in Singapore's populations of the broadcast spawning sea star *Protoreaster nodosus*

**DOI:** 10.1098/rsos.160253

**Published:** 2016-08-17

**Authors:** Y. C. Tay, M. W. P. Chng, W. W. G. Sew, F. E. Rheindt, K. P. P. Tun, R. Meier

**Affiliations:** 1 Department of Biological Sciences, National University of Singapore, 14 Science Drive 4, Singapore 117543, Republic of Singapore; 2 Centre for Liveable Cities, Ministry of National Development, The URA Centre, 45 Maxwell Road no. 07-01, Singapore 069118, Republic of Singapore; 3 DHI Water and Environment (S) Pte Ltd, 1 Cleantech Loop, Cleantech One, no. 03-05, Singapore 637141, Republic of Singapore; 4 National Parks Board, 1 Cluny Road, Singapore 259569, Republic of Singapore; 5 Lee Kong Chian Natural History Museum, Department of Biological Sciences, National University of Singapore, Singapore, Republic of Singapore

**Keywords:** *Protoreaster nodosus*, genetic diversity, ddRADseq, broadcast spawner, species resilience

## Abstract

The Coral Triangle is widely considered the most important centre of marine biodiversity in Asia while areas on its periphery such as the South China Sea, have received much less interest. Here, we demonstrate that a small population of the knobbly sea star *Protoreaster nodosus* in Singapore has similarly high levels of genetic diversity as comparable Indonesian populations from the Coral Triangle. The high genetic diversity of this population is remarkable because it is maintained despite decades of continued anthropogenic disturbance. We postulate that it is probably due to broadcast spawning which is likely to maintain high levels of population connectivity. To test this, we analysed 6140 genome-wide single nucleotide polymorphism (SNP) loci for Singapore's populations and demonstrate a pattern of near panmixia. We here document a second case of high genetic diversity and low genetic structure for a broadcast spawner in Singapore, which suggests that such species have high resilience against anthropogenic disturbances. The study demonstrates the feasibility and power of using genome-wide SNPs for connectivity studies of marine invertebrates without a sequenced genome.

## 1. Introduction

The Coral Triangle is widely acknowledged as the origin and centre of high marine biodiversity [1,2], and has therefore attracted much attention and research. Recently, there has been much debate as to the evolutionary origin of its high biodiversity (reviewed in [1,3]) and biologists are now starting to appreciate the important contributions from peripheral areas. For instance, Bowen *et al*. [3] suggested a biodiversity feedback model in which peripheral populations are important in supporting the processes that maintain high diversity within the Coral Triangle. The South China Sea, which is on the western boundary of the Coral Triangle, is part of the biodiversity-rich periphery [4] that contributes to the area (e.g. [5]), but has received much less attention than the Coral Triangle. Fortunately, the many studies on fauna in the Coral Triangle (e.g. [6–9]) provide excellent background information for comparing patterns of diversity with its peripheral areas such as the South China Sea. A good example is the widespread knobbly sea star, *Protoreaster nodosus*, which was the subject of a previous phylogeographic study that documented a continuous genetic landscape across approximately 2000 km of the archipelagic Indonesian extent of the Coral Triangle from Karimunjawa to Raja Ampat [10].

Although not considered endangered at a global scale, knobbly sea stars are listed as endangered in Singapore waters due to their restricted distribution [11] within a few seagrass and shallow sandy intertidal habitats [12]. Rapid economic development over the past few decades has led to significant fragmentation of Singapore's coastal and marine habitats, mainly due to conversion of coastal habitats and land reclamation [13]. Here, we compare the population genetic diversity of the highly impacted knobbly sea star populations in Singapore at the southern end of the South China Sea, with the less impacted populations within the Coral Triangle in an effort to assess the status of Singapore's populations while allowing for the identification of anthropogenic signatures. Reduced genetic diversity and higher rates of linkage disequilibrium, for example, can indicate a recent population bottleneck, and diversifying selection can suggest local adaptive forces [14].

With anticipated near- and long-term changes to Singapore's coastal profile articulated in the country's latest strategic land use and transportation plans [15], there is a need to understand the current status of Singapore's marine biodiversity, which will facilitate resource management agencies in identifying conservation priorities while providing a baseline for future comparisons. Our current study mirrors a similar study of *Platygyra sinensis*, a scleractinian coral species in Singapore that suggested no significant anthropogenic impact on the genetic diversity or connectivity of this broadcast spawner [16] despite substantial reef loss and fragmentation. Given the similarity of the reproductive characteristics of *P. nodosus* to broadcast-spawning corals, namely high reproductive capacity as a broadcast spawner and long planktonic larval duration (PLD) of two to four weeks [17], genetic connectivity and diversity of Singapore's *P. nodosus* populations could also be expected to be high, but the population dynamics might differ between coral reef and seagrass-dependent species, especially because Singapore's seagrass beds occupy a much smaller area and are more fragmented [18]. Therefore, in this paper we aim to (i) compare the population genetics of *P. nodosus* in Singapore waters with those in the Indonesian region of the Coral Triangle to determine if genetic diversity has been impacted in populations of the former and (ii) investigate the fine-scale genetic structure of Singapore's *P. nodosus* populations using thousands of genome-wide single nucleotide polymorphism (SNP) markers, which are capable of detecting low levels of genetic structure not reflected by the traditional cytochrome oxidase I (COI) genetic marker used in previous studies of Coral Triangle populations (e.g. [19–22]).

## 2. Material and methods

### 2.1. Tissue sampling

**Figure 1.**
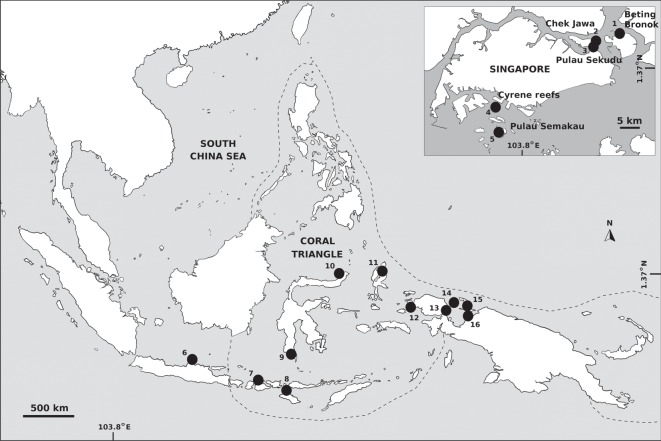
Locations of *Protoreaster nodosus* populations from Singapore and Crandall *et al*. [10] analysed in this study (numbered black circles). Corresponding specific site names are in the electronic supplementary material, table S1. Sampling locations in Singapore are presented in the inset map. Boundaries of the Coral Triangle are highlighted with a dotted line.

Five to ten tube feet were collected from 80 individuals of *Protoreaster nodosus* from five locations in Singapore (‘north’: Pulau Sekudu, Chek Jawa and Beting Bronok; ‘south’: Cyrene reefs and Pulau Semakau; figure 1) from January to July 2013, preserved in molecular grade ethanol and stored at −20°C. Photo-identification vouchers of the aboral surface of individuals [23] were taken prior to release of the animals, to prevent repeat genetic sampling of the same individual.

### 2.2. Mitochondrial analyses

Genotyping was performed using the tRNAasn42F and ValvaCOI-770R primers [10] for the mitochondrial cytochrome oxidase subunit-I (COI). PCR conditions were optimized to bypass DNA extraction and conduct PCR directly on the preserved tissue [24]. Tissue samples were placed directly in 25 µl reactions containing 1× reaction buffer, 0.8 mM total dNTPs, 0.4 µM each primer and 0.8 µl of a generic Taq polymerase. Cycling parameters were a step-up protocol of 95°C (3 min), 10 cycles of 94°C (30 s), 58°C (30 s) and 72°C (60 s), followed by 20 cycles of 94°C (30 s), 60°C (30 s) and 72°C (60 s), and a final extension of 72°C (5 min). PCR products were purified using SureClean™ (Bioline) following manufacturer's instructions, and sequenced using BigDye™ v. 3.1 (Applied Biosystems Inc.) on an ABI Avant 3130xl Genetic Analyzer. Sequence chromatograms were checked for quality, assembled and translated to check for stop codons in Sequencher™ v. 4.5 (Gene Codes Corporation).

COI sequence data were also obtained for *P. nodosus* populations from the Coral Triangle [10] (figure 1). All sequences were aligned and trimmed to 710 bp using MEGA v. 6 [25]. After removing samples with significant portions of missing terminal sequences and small populations with less than 10 individuals, a total of 363 individuals were analysed (electronic supplementary material, table S1). Haplotype and nucleotide diversities were calculated using DnaSP [26]. Other population genetic parameters were computed using Arlequin v. 3.5 [27]: (i) pairwise population differentiation statistics (*Φ*_ST_ values) were calculated with 10 000 pairwise permutations for which the *p*-values were subject to the Benjamini–Hochberg procedure to correct for multiple comparisons [28], (ii) a mismatch distribution analysis was conducted under the model of sudden demographic expansion using 10 000 bootstrap replicates, (iii) tests for neutrality were evaluated with Tajima's *D* [29,30] and Fu's *F*_S_ [31], and (iv) a minimum spanning network was computed for all 363 individuals with 10 000 permutations. The network was visualized in HapStar [32]. To account for possible bias in genetic diversity estimates (number of haplotypes and haplotype diversity) due to sample size variations, each population was rarefied 30 times to match one of the smallest sample sizes of 15 for Numfor [33]. Four sampling sites that had fewer than 10 individuals were excluded.

### 2.3. ddRADseq library preparation

DNA extracts were prepared from two to four tube feet following the manufacturer's protocol of the Biospin tissue genomic DNA extraction kit. Extracted DNA samples were quantified using NanoDrop 1000 and assessed for quality on a 1% agarose gel. In total, 36 samples were selected from across the three main sampling localities, Pulau Sekudu, Pulau Semakau and Cyrene reefs, based on DNA quality. A double-digest restriction enzyme associated DNA sequencing (ddRADseq) library was then prepared from these extracts for genome-wide SNP analyses, using the adapters and PCR primer pairs in Peterson *et al*. [34] (refer to electronic supplementary material, table S2, for samples and corresponding barcodes and indexes). A total of 100 ng DNA from each sample was simultaneously double-digested with restriction enzymes and ligated to adapters in duplicate 13 µl reactions at 37°C for 3.5 h. Each reaction contained 5 U EcoRI-HF® (NEB), 1 U MspI, 80 U T4 DNA ligase, 1× T4 DNA ligase buffer, 50 mM NaCl, 0.05 mg ml^−1^ bovine serum albumin and 3.85 µM of each adapter. The duplicate digestion–ligation reactions were pooled and size-selected using Sera-Mag™ Magnetic SpeedBeads™ Carboxylate-Modified suspended in an 18% PEG-8000 (w/v) buffer (1 M NaCl, 10 mM Tris-HCl, 1 mM EDTA, pH 8). Briefly, 18 µl per sample was subjected to size-selection with sequential bead : DNA ratios of 0.78× and 0.95× to extract 250–600 bp DNA-adapter fragments, washed with 85% ethanol and resuspended in 20 µl molecular grade water. Triplicate PCRs were used to amplify 2 µl of the size-selected, adapter-ligated DNA fragments in a 10 µl reaction containing 1× Q5 reaction buffer, 200 µM each dNTP, 0.2 µM each PCR primer and 0.2 U Q5® High-Fidelity DNA polymerase. PCR cycling conditions were as follows: 98°C for 30 s, two cycles of 98°C for 10 s, 55°C for 30 s and 72°C for 1 min, 18 cycles of 98°C for 10 s and 68°C for 1 min, followed by a final extension at 72°C for 2 min. Size-selection was repeated using the same bead : DNA ratios as before, to ensure clean products of the desired fragment size. Purified DNA libraries for 36 *P. nodosus* samples were quantified using the Qubit® 2.0 Fluorometer, pooled in equal proportions, and sequenced on a single Illumina HiSeq lane (100 bp paired-end, occupying approx. 65% of the sequencing run).

### 2.4. ddRADseq analyses

Read quality of the raw sequencing data was assessed using FastQC v. 0.11.2 [35]. Data processing using STACKS v. 1.24 [36] was as follows: (i) sample reads were demultiplexed, trimmed to 94 bp based on quality scores for Read 1 (electronic supplementary material, figure S1), filtered for low quality bases and reads with a sliding window score limit of Phred 20 in *process_radtags*. The rescue barcodes option was enabled. Only Read 1 of the paired-end data was used for subsequent radtag assemblies and SNP calling, reducing the inclusion of erroneous chimera sequences in subsequent analyses, which may have formed during the DNA ligation step of the RAD DNA library preparation. Furthermore, any read containing restriction recognition sites of EcoRI or MspI, which are potential chimera junctions, was removed using a custom bash script. The subsequent SNP-calling pipeline was repeated for both datasets, filtered and not filtered for potential chimeras, to assess the degree and impact of potential chimera formation on the results. One sample (TP12) was excluded from subsequent steps due to low amounts of data retrieved. (ii) For each sample, reads were sorted into ‘stacks’ of at least five identical reads using the *ustacks* module. A sensitivity analysis of the mismatch thresholds (*M*) for merging of stacks was conducted with 1, 2, 4, 7 and 14 bp mismatches between stacks. The removal and deleveraging algorithms were enabled to remove highly repetitive stacks and resolve over-merged stacks. (iii) A catalogue of radtag loci was created with loci from across all samples using *cstacks* with the same number of mismatches allowed between stacks during step (ii) as the number of mismatches between loci to generate the catalogue. (iv) Stacks from each sample were matched against the radtag locus catalogue to determine the allelic state in the *sstacks* module. (v) The *populations* module was then used to filter and retain the first SNP per radtag locus with a stack depth of at least 30 and not more than 20% missing data, and calculate basic population statistics such as nucleotide diversities and heterozygosities. Several trial runs were conducted under the exclusion of one to several samples with little data. Two samples, SE20 and TP14, were eventually excluded from further analyses because the number of SNPs called dropped below 100 when these samples were included. The final dataset consisted of 33 samples. A minor allele filter was also applied to remove alleles present in only one (MAF > 0.06) of these 33 individuals. Preliminary sample clustering analyses suggested little differences in the final population structuring patterns across different parameter setting combinations (electronic supplementary material, figure S2). We chose to focus on a mismatch threshold of four as the number of SNP loci mined increased to almost a plateau for most datasets from four to seven (electronic supplementary material, figure S3). Effects of minimum stack depths on population genetic structure were then explored (mismatch thresholds fixed at 4) using initial stack depths of 3 and 5, and final stack depths of 20 and 10 (electronic supplementary material, figure S4). Because the version of STACKS used here did not consider indels which may lead to over-splitting of loci, SNP calls were also made using pyRAD v. 3.0.65 [37] at several settings (refer to the electronic supplementary material).

Input files for downstream analyses were formatted using PGDSpider v. 2.0.5.1 [38]. Arlequin was used to screen for loci that were found to deviate from Hardy–Weinberg equilibrium (HWE). Identification of loci possibly under selection was performed using Bayescan v. 2.1 [39], with a burn-in of 5 × 10^4^ followed by 1 × 10^5^ iterations, and all other parameters left as default. At false discovery rate (FDR) levels of 0.05, 0.1 and 0.2, loci were determined as under directional (diversifying) selection when positive alpha values were encountered, and under balancing or purifying selection (background) when zero or negative *α* values were encountered [39,40]. Loci found to deviate from Hardy–Weinberg equilibrium, or under diversifying selection were removed from subsequent analyses. Bayesian clustering analyses were performed in STRUCTURE v. 2.3.4 for up to five genetic clusters (*K*) considering correlated allele frequencies in the admixture model and without using sampling locations as priors [41–43]. Preliminary trials were run to achieve a burn-in that allowed for convergence of *α*. StrAuto [44], which is a Python program that streamlines and automates multiple iterations of STRUCTURE runs in its Unix command line version, was used to automate 10 iterations of a 6 × 10^5^ burn-in followed by 1 × 10^5^ MCMC iterations for data collection. The optimal *K* was determined using STRUCTURE HARVESTER [45,46]. To account for variations across the 10 iterations of STRUCTURE runs, the *Greedy* algorithm in CLUMPP v. 1.1 [47] was used, and the resulting barplots were constructed using DISTRUCT v. 1.1 [48]. Pairwise population differentiation statistics (*G*′_ST_ [49], *D*_Jost_ [50], *F*_ST_ [51]) were calculated with 100 bootstrap replicates (across loci) using the *fastDivPart* function in the package diveRsity [52] that is available in R [53]. The discriminant analysis of principal components (DAPC) which identifies clusters of genetically related individuals without relying on any population genetic model, and is therefore free of HWE or linkage disequilibrium assumptions [54], was performed in the R package adegenet v. 2.0.0 [55].

## 3. Results

**Figure 2.**
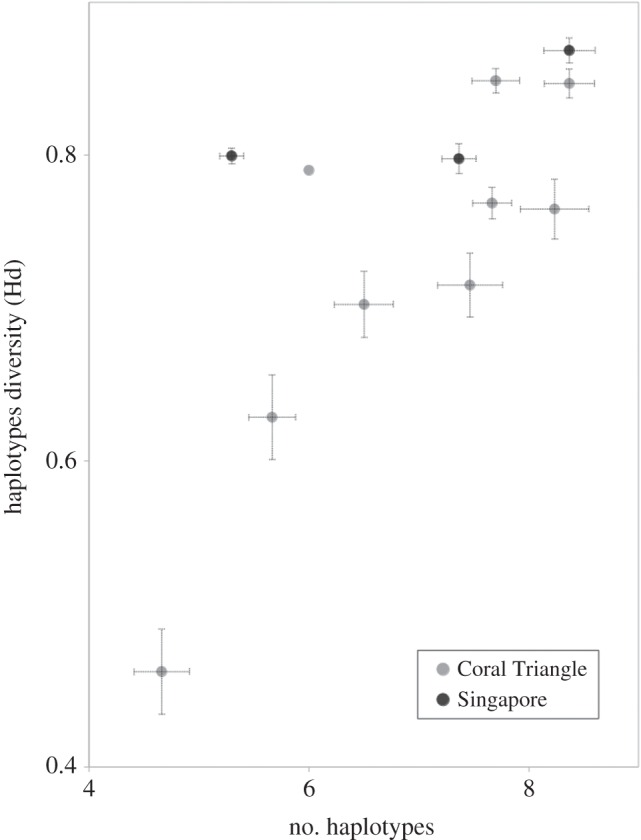
Average genetic diversities of three sampling localities in Singapore and nine from within the Coral Triangle based on COI data. Different sample sizes were accounted for by 30 sets of random subsamples of 15 individuals per sampling locality. Number of haplotypes per site are represented on the *x*-axis, haplotype diversities on the *y*-axis. Standard error bars are indicated. Specific site diversity and standard error values are in the electronic supplementary material, table S1.

**Figure 3.**
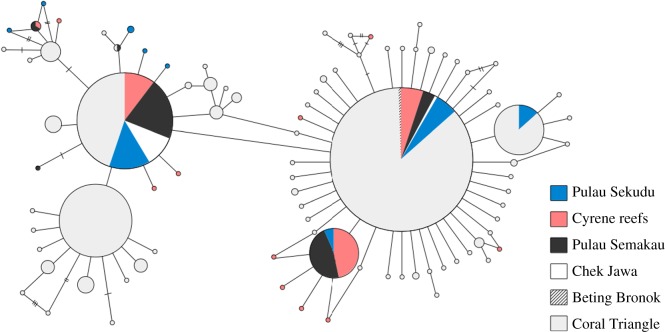
Minimum spanning network for *Protoreaster nodosus* populations in Singapore and the Coral Triangle based on COI sequence data. All samples from within the Coral Triangle are grouped together as the lightest shade of grey. Each haplotype is represented by one circle and separated by one mutational step, unless indicated by additional hatch marks. Diameters of circles are proportionate to the frequency of each haplotype occurrence, ranging from 1 to 29, except for the most common haplotype (178) which was scaled down.

**Figure 4.**
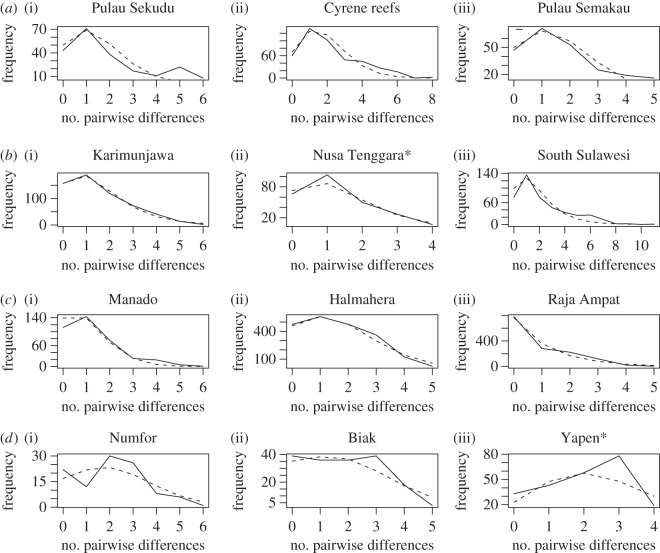
Frequency distribution of the number of pairwise sequence differences among individuals from each sampling locality in Singapore ((*a*)(i–iii)), and across the Coral Triangle ((*b*–*d*)(i–iii)). *p-value of SSD test for goodness-of-fit with the model of sudden population expansion was significant at the 0.05 level.

Genetic diversity of Singapore's *P. nodosus* populations as assessed by COI haplotype diversity was high; rarefied haplotype diversity of the three main populations at Pulau Sekudu, Cyrene reefs and Pulau Semakau in Singapore (0.798–0.869) fell in the upper end of the spectrum of the Coral Triangle (figure 2; electronic supplementary material, table S1), and 18 of the 91 haplotypes were unique to Singapore (GenBank accession numbers KU896219–KU896296)—most of which were found at Cyrene reefs (figure 3). Mismatch distribution analysis results suggest that most of the locations investigated in this study have undergone a recent population expansion; both tests of goodness of fit from the model of instantaneous demographic expansion were mostly non-significant at the 0.05 level (table 1), which indicates that there is no significant difference (i.e. a good fit) between the observed data and data expected under a model of demographic expansion. Plots of pairwise differences were also roughly unimodal and smooth (figure 4), suggesting an excess of low frequency mutations acquired during population expansion, compared with stationary populations that have been subject to more genetic drift and tend to exhibit multimodal distributions [56–58]. Populations at Nusa Tenggara and Yapen, however, differed significantly from the model of recent population expansion as indicated by the sum of squared deviations (SSD), but not the Raggedness index. The many unique haplotypes and strongly negative Fu's *F* statistic and Tajima's *D* (table 1; electronic supplementary material, table S1) are also consistent with a recent population expansion at most sites except Pulau Semakau and Numfor.

**Table 1.** Results of the mismatch distribution analysis and tests for neutrality, for *Protoreaster nodosus* populations at each sampling location, based on COI data. Statistically significant values are highlighted in italics.

		*τ* (low bound; upper bound)	*θ*0 (low bound; upper bound)	*θ*1 (low bound; upper bound)	SSD (*p*-value)	Raggedness index (*p*-value)	Tajima's *D* (*p*-value)	Fu's *F*s (*p*-value)
3	Sekudu	1.498 (0.436; 3.246)	0.039 (0.000; 0.932)	20.064 (1.443; 166.629)	0.0164 (*p* = 0.119)	0.0619 (*p* = 0.195)	−1.52 (*p* = 0.049)	−*3.36* (*p = 0.018*)
4	Cyrene	1.863 (0.828; 3.121)	0.000 (0.000; 1.053)	94.376 (2.238; 140.314)	0.0074 (*p* = 0.097)	0.0512 (*p* = 0.072)	*−1.94* (*p = 0.010*)	*−8.01* (*p < 0.001*)
5	Semakau	1.811 (0.457; 4.018)	0.000 (0.000; 1.016)	9.778 (1.189; 164.78)	0.0044 (*p* = 0.510)	0.0372 (*p* = 0.638)	−0.64 (*p* = 0.295)	−0.46 (*p* = 0.401)
6	Karimunjawa	0.828 (0.578; 4.258)	0.752 (0.000; 0.703)	23.346 (1.466; 35.501)	0.0006 (*p* = 0.881)	0.0281 (*p* = 0.791)	*−2.12* (*p = 0.003*)	*−11.75* (*p < 0.001*)
7	Nusa Tenggara	0.875 (0.000; 0.625)	0.450 (0.000; 0.000)	7158.133 (4963.143; 7148.133)	*0.0056* (*p = 0.000*)	0.0806 (*p* = 1.000)	−1.4 (*p* = 0.0750)	*−3.7* (*p = 0.006*)
9	South Sulawesi	0.781 (0.662; 5.219)	1.013 (0.000; 1.118)	3431.002 (1.779; 35.892)	0.0088 (*p* = 0.284)	0.0466 (*p* = 0.199)	*−2*.*09* (*p = 0*.*006*)	*−6*.*06* (*p = 0*.*002*)
10	Manado	1.000 (0.371; 2.945)	0.000 (0.000; 0.838)	3407.185 (0.889; 714.695)	0.0068 (*p* = 0.390)	0.0596 (*p* = 0.311)	*−1*.*98* (*p = 0*.*007*)	*−5*.*87* (*p < 0*.*001*)
11	Halmahera	1.996 (0.592; 4.529)	0.011 (0.000; 0.990)	5.831 (1.486; 118.33)	0.0014 (*p* = 0.778)	0.0231 (*p* = 0.891)	*−2*.*37* (*p < 0*.*001*)	*−27*.*79* (*p < 0*.*001*)
12	Raja Ampat	3.174 (0.703; 7.385)	0.000 (0.000; 0.197)	0.873 (0.544; 26.577)	0.0054 (*p* = 0.857)	0.1339 (*p* = 0.598)	*−2*.*26* (*p = 0*.*001*)	*−11*.*37* (*p < 0*.*001*)
14	Numfor	2.754 (1.215; 5.877)	0.000 (0.000; 1.269)	7.231 (1.99; 160.982)	0.0217 (*p* = 0.331)	0.0720 (*p* = 0.357)	−1.24 (*p* = 0.109)	−0.72 (*p* = 0.306)
15	Biak	2.674 (1.068; 5.658)	0.000 (0.000; 1.162)	4.746 (1.728; 137.403)	0.0059 (*p* = 0.729)	0.0237 (*p* = 0.958)	−1.28 (*p* = 0.099)	*−3*.*9* (*p = 0*.*007*)
16	Yapen	2.547 (1.244; 4.199)	0.000 (0.000; 1.587)	34.101 (3.027; 254.102)	*0*.*0217* (*p = 0*.*031*)	0.0856 (*p* = 0.053)	−1.13 (*p* = 0.132)	*−4*.*13* (*p = 0*.*008*)

Excluding three data-deficient samples, 82.0 ± s.d. 3.5% of the barcoded reads from the Illumina HiSeq sequencer remained after quality filtering (1.7 to 7.1 × 10^6^ reads per sample). Inspection of the raw sequence files revealed that this high drop-out rate was largely due to the presence of an N base call at the second position of the barcode region. Of these, 93.0 ± s.d. 1.7% were retained per sample after filtering for potential sites of chimera formation. The sensitivity analysis of mismatch thresholds for calling SNPs showed that varying the mismatch threshold had little effect on results. Log-likelihood score profiles of the assembled stacks (good likelihood ratios are close to zero, while loci with highly negative log likelihood scores tend to have low coverage or high sequencing errors [36]) displayed gradual negative displacement with increasing mismatch thresholds (*M*) although the distributions for *M* = 1 and *M* = 2 were similarly close to zero (electronic supplementary material, figure S5). *F*_IS_ distributions across the mismatch thresholds also displayed a similar pattern of minimal effect (electronic supplementary material, figure S6*a–c*). Applying a minor allele frequency filter of requiring alleles to be present in at least two or more individuals (MAF > 0.06), however, resulted in a shift in peak away from *F*_IS_ = 0 and increase in the number of loci at *F*_IS_ = 1 (electronic supplementary material, figure S6*a–c*), which was similar to the effect of using the *rxstacks* catalogue correction module in STACKS (preliminary trials, data not presented). A similar effect was also reported in [59]. All in all, 1431–6140 SNP loci were called across the various parameter combinations tested (electronic supplementary material, figure S3, shows SNP numbers for mismatch threshold sensitivity analysis), but results were similar across all combination sets. Here, we present the SNP analyses with 6140 SNP loci called using a mismatch threshold of 4, final stack depth of 10 and with the minor allele frequency filter of more than 0.06.

Only less than or equal to three loci were identified as outliers in Bayescan (electronic supplementary material, figure S7*b*). Considering only loci that were polymorphic, genetic diversities across the three main sampling sites in Singapore were similar with minimal variation (table 2). Compared with previous studies of marine fish and a sea anemone that also used STACKS to call genome-wide SNPs, the genetic diversity of Singapore's *P. nodosus* populations are moderately high (*H*_obs-SIN_ 0.298–0.305, table 2 versus *H*_obs_ 0.023–0.402, [20,60,61]), although comparability across such datasets needs to be viewed with caution when based on different SNP sets.

**Table 2.** Summary of population genetic statistics considering the variant positions among the 6392 ddRADseq SNP loci called by STACKS, when 20% missing data was allowed. *N*, number of individuals; *p*, average frequency of the major allele; *H*_obs_, average observed heterozygosity per locus; *H*_exp_, average expected heterozygosity per locus; *π*, average nucleotide diversity and *F*_IS_, the average Wright's inbreeding coefficient.

	*N*	*p*	*H*_obs_	*H*_exp_	*π*	*F*_IS_
Pulau Sekudu	11.7	0.789	0.268	0.294	0.307	0.11
Pulau Semakau	10.3	0.792	0.257	0.291	0.305	0.13
Cyrene Reefs	9.4	0.793	0.258	0.289	0.305	0.13

**Figure 5.**
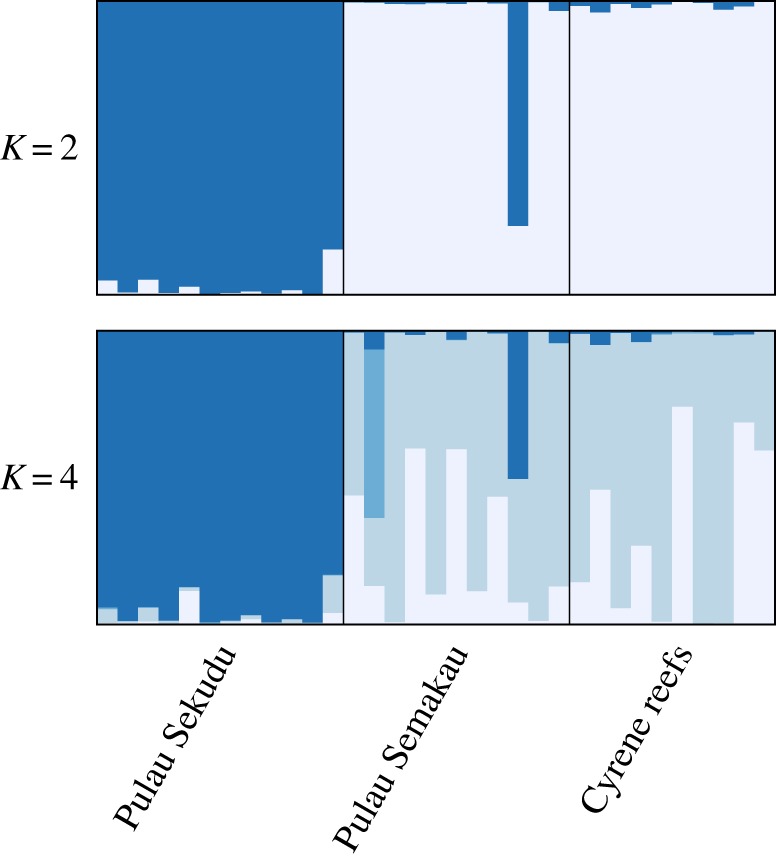
STRUCTURE barplots at *K* = 2 and *K* = 4 calculated over 10 iterations (dataset of 6140 SNPs; *M* = 4, *m* = 5,10, MAF > 0.06). Each bar depicts the genotype assignment for each individual. Barplots at other SNP-calling parameter sets showed similar profiles (data not shown).

**Figure 6.**
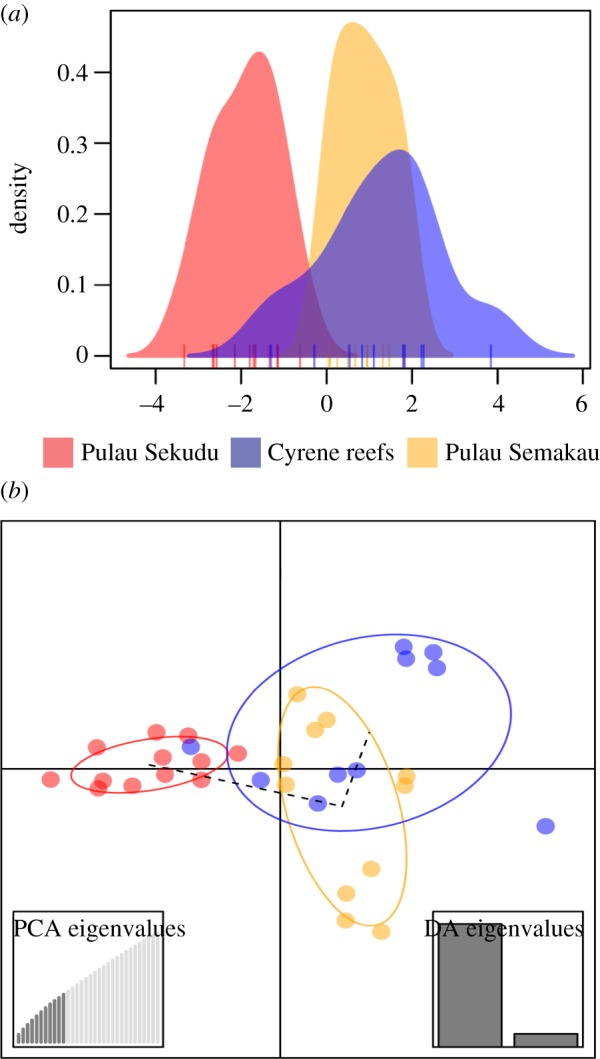
DAPC plots when one (*a*) and two (*b*) discriminant functions were retained (dataset of 6140 SNPs; *M* = 4, *m* = 5,10, MAF > 0.06). Similar cluster profiles were observed for other SNP-calling parameter sets (electronic supplementary material, figures S7–S9).

Only very subtle genetic structure (statistically non-significant) was detected among Singapore's *P. nodosus* populations in both the mitochondrial COI and ddRADseq SNP data. Of the 22 COI haplotypes found in Singapore waters, the three most common haplotypes were detected at three of the five locations (figure 3). Pairwise *Φ*_ST_ was highest between the population at Pulau Sekudu and Cyrene reefs in Singapore (*Φ*_ST_ = 0.05, significant at the 0.05 level but not after performing the Benjamini–Hochberg correction, table 3). This weak genetic structure between Pulau Sekudu, which is located north of mainland Singapore, and the sampling locations to the south of Singapore was consistent across the analyses of thousands of genome-wide SNP loci called with different parameter combinations. Although none of the pairwise *F*_ST_ values across all SNP datasets were statistically significant, the pairwise *F*_ST_ value between Pulau Semakau and Cyrene reefs was almost fivefold smaller than pairwise *F*_ST_ values between Pulau Sekudu and the two southern sites (table 4, 95% confidence intervals were all inclusive of zero). Furthermore, the same two genetic clusters were identified by both the STRUCTURE and STRUCTURE HARVESTER analyses (figure 5; electronic supplementary material, figure S8), and DAPC scatter plots (figure 6; electronic supplementary material, figures S2 and S4). SNP loci obtained using pyRAD also suggested the same genetic clusters (electronic supplementary material, figure S9). At a larger scale, COI sequence data detected low but significant levels of genetic differentiation between the *P. nodosus* populations in Singapore, with populations approximately 2800 km eastwards within the Coral Triangle until Halmahera (0.05 ≤ *Φ*_ST_ ≤ 0.11, table 3). Similar to what was reported in Crandall *et al*. [10], a significant genetic break was found at the eastern-most cluster of *P. nodosus* populations beyond Raja Ampat (0.09 ≤ *Φ*_ST_ ≤ 0.27, table 3).

**Table 3.** Pairwise Φ_ST_ comparisons based on COI sequence data, computed in Arlequin. Φ_ST_ values below the diagonal, while *p*-values are above. Significant values after the Benjamini–Hochberg correction in italics (*p* < 0.05), and indicated with an asterisk when *p* < 0.01. Sample sizes of each population are given in parentheses.

		Pulau Sekudu	Cyrene Reefs	Pulau Semakau	Karimunjawa	Nusa Tenggara	South Sulawesi	Manado	Halmahera	Raja Ampat	Numfor	Biak	Yapen
3	Pulau Sekudu (*n* = 21)		0.03287	0.287	0.10	0.10949	0.23295	0.13	0.06	0.00	0.00	0.03	0.00
4	Cyrene reefs (*n* = 30)	0.05		0.37	0.00	0.00	0.00	0.00	0.00	0.00	0.00	0.00	0.00
5	Pulau Semakau (*n* = 22)	0.01	0.00		0.00	0.00	0.01	0.01	0.00	0.00	0.00	0.01	0.00
6	Karimunjawa (*n* = 35)	*0*.02*	*0*.06*	0.09		0.64	0.80	0.66	0.51	0.11	0.00	0.00	0.00
7	Nusa Tenggara (*n* = 23)	*0*.03*	*0*.08*	0.11	−0.01		0.72	0.36	0.14	0.01	0.00	0.03	0.00
9	South Sulawesi (*n* = 30)	*0*.01*	*0*.*05*	0.06	−0.01	−0.01		0.54	0.11	0.01	0.00	0.01	0.00
10	Manado (*n* = 28)	*0*.02*	*0*.*06*	0.08	−0.01	0.00	0.00		0.79	0.09	0.00	0.02	0.00
11	Halmahera (*n* = 64)	*0*.02*	*0*.06*	0.07	0.00	0.01	0.01	−0.01		0.10	0.00	0.02	0.00
12	Raja Ampat (*n* = 54)	*0*.11*	*0*.11*	*0*.18*	0.01	*0*.*05*	*0*.*03*	0.02	0.01		0.00	0.00	0.00
14	Numfor (*n* = 15)	*0*.20*	*0*.27*	*0*.23*	*0*.30*	*0*.30*	*0*.24*	*0*.30*	*0*.26*	*0*.43*		0.07	0.34
15	Biak (*n* = 19)	*0*.06*	*0*.*09*	0.09	*0*.*09*	0.08	*0*.*06*	*0*.*08*	*0*.*05*	*0*.14*	0.07		0.11
16	Yapen (*n* = 22)	*0*.17*	*0*.23*	*0*.19*	*0*.24*	*0*.24*	*0*.20*	*0*.24*	*0*.20*	*0*.35*	0.00	0.04

**Table 4.** Pairwise *F*_ST_ (W&C) comparisons calculated based on 6140 ddRADseq SNP loci in the R package diveRsity. Bias corrected 95% confidence intervals for each pairwise *F*_ST_ are indicated in brackets above the diagonal. *G*′_ST_, *D*_Jost_ were also calculated, but all three indices gave insignificant pairwise comparisons, so only the *F*_ST_ (W&C) values are presented here. Pairwise *F*_ST_ values were also calculated for all SNP-calling parameter sets (data not shown), and all runs gave similar patterns.

	Cyrene reefs	Pulau Semakau	Pulau Sekudu
Cyrene reefs		(−0.030, 0.037)	(−0.023, 0.042)
Pulau Semakau	−0.0017		(−0.018, 0.039)
Pulau Sekudu	0.0066	0.0066	

## 4. Discussion

The most surprising finding is the remarkably high level of genetic diversity in Singapore's *P. nodosus* population in relation to those within the Coral Triangle, despite the many anthropogenic impacts on Singapore's marine environment [62] and the small size of the remaining habitats (sum of three largest seagrass meadows in Singapore = 33.7 ha [18]). This could be due to Singapore's central location between the Indian Ocean and the western tropical Pacific, which lies in the middle of its geographical range [63], but it also suggests high genetic resilience of this species that is probably due to its broadcast-spawning characteristics. While the Coral Triangle is widely acknowledged as a hotspot and origin of marine biodiversity (e.g. [1,2]), our analyses reveal similarly high genetic diversity in Singapore's populations, which lie just outside the Coral Triangle and at the southern end of the South China Sea. Our finding of high genetic diversity is in agreement with earlier reports on high species diversity within the South China Sea [64] and highlights the importance of the South China Sea for the maintenance of genetic diversity in marine invertebrates in tropical Asia [4]. Despite the low global conservation priority of *P. nodosus*, we show that Singapore's *P. nodosu*s populations can be important contributors to the global gene pool, with 18 COI haplotypes unique to a small area.

A near-panmictic genetic landscape was found for most of the *P. nodosus* populations (table 3), extending approximately 2800 km eastwards from Singapore into the Coral Triangle. At a smaller scale within Singapore waters, low and statistically insignificant pairwise *F*_ST_ values also indicated near panmixia. Widespread dispersal and high connectivity is generally expected for a broadcast-spawning species with a long larval planktonic duration (e.g. [65,66], and see [67]) such as *P. nodosus* [17]*.* This is especially because the spawning period of *P. nodosus* is expected to coincide with synchronous mass coral spawning in Singapore [12,68], and high connectivity and high levels of larval exchange have been predicted for broadcast-spawning coral larvae among Singapore's southern islands during this period [69]. High levels of connectivity among populations typically increase effective population sizes, and could have contributed to relatively high levels of genetic diversity [70–72] observed here. However, while PLD plays a major role in determining connectivity, other factors have also been shown to affect the connectivity patterns so that PLD is not necessarily a good predictor of connectivity (e.g. see [73–78]). Also, just 1–10 migrants per generation can be sufficient to minimize heterogeneity among populations [79] and can lead to a pattern of apparent panmixia. It is thus difficult to discern based on genetic structure alone whether the apparent high connectivity is a result of ongoing gene flow or a genetic signature of past dispersal events (reviewed in [80]). Preliminary hydrodynamic modelling of the dispersal of *P. nodosus* larvae within Singapore waters [81] suggests high rates of ongoing larval exchange especially across the Southern Islands, and less pronounced exchange between sites to the north (Johor Strait) and south (Singapore Strait) of mainland Singapore. This may have contributed to the observed subtle genetic structure between ‘populations’ to the north and south. Crandall *et al*. [10] previously also demonstrated fine-scale structure across a much shorter 13 km stretch of coastal ocean despite using only mitochondrial sequences instead of SNP data which provides better resolution (e.g. [19–22]), which could be due in part to the positive geotactic behaviour approximately 2 days after attaining motility in larvae of the knobbly sea stars [17] that reduces the window of opportunity for long-distance dispersal by the currents. Other factors that could have contributed to the fine-scale genetic structure in this species in Singapore include anthropogenic impacts such as (i) the high shipping traffic [62] across more than 30 km of coastal waters, (ii) coastal pollution from runoff and effluent from a highly urbanized terrestrial environment, which has been suggested to be an effective barrier to larval dispersal [82], and/or (iii) the lack of many suitable habitats [18] for larval settlement and growth into reproductive adults. The low genetic divergence and lack of loci under diversifying selection, however, suggest that the possible anthropogenic impacts on this species in Singapore, if any, are still minimal.

Although neither population genetic differentiation estimates for the mitochondrial nor SNP datasets were statistically significant, the relative estimates for north–south comparisons were consistently higher than within the Southern Islands (an almost fivefold difference in pairwise *F*_ST_ values in the SNP dataset, tables 3 and 4). The robustness of relative *F*_ST_ values that reflect relationships or clustering patterns have also been shown despite changes in absolute values with different mismatch parameter settings during SNP loci clustering [83]. Nevertheless, the absolute *F*_ST_ values obtained in this study should be cautiously interpreted because the inclusion of only a subset of 10–12 samples per population in SNP analyses may have resulted in an over-estimation of pairwise *F*_ST_ values (sample sizes smaller than 4–6; [84]). However, the use of a large number of SNP loci (more than 1000, this study used 1431–6140) coupled with the use of the Weir and Cockerham estimate of genetic differentiation [51] should still allow for accurate detection of even low levels of population genetic differentiation [84]. A false-positive detection of genetic structure based on *F*_ST_ values in this study is also unlikely because the *F*_ST_ values in this study are already low and not significant. Furthermore, the more than 10-fold difference in pairwise *F*_ST_ values found for north–south comparisons, compared to within the Southern islands (table 4), was also supported by STRUCTURE and DAPC analyses. DAPC analyses are less likely to be biased due to the small sample sizes because they are not based on any population genetic model assumptions such as HWE or linkage disequilibrium [54]. Hence, we are confident that the observed low level of genetic structuring is not an artefact of small sample size.

This is the second case where we find high genetic diversity and apparent connectivity in a broadcast-spawning marine species that lives in the heavily impacted marine environment of Singapore [62]. The first was the scleractinian coral *Platygyra sinensis* [16]. These studies support the contention that broadcast spawners may have higher resilience to anthropogenic disturbances, which should facilitate their survival in human-impacted habitats, and concur with the assessment by Jones *et al*. [85] that highly connected systems grant populations greater resilience to perturbations due to enhanced recovery rates via external larval seeding. Conversely, isolated reef systems with broadcast spawners have been shown to display slower recovery rates when the systems rely on the reproductive success of the few remaining survivors [86]. In some cases, one good population may suffice to seed impacted sites upon recovery from disturbance. Indications of recent population expansion were found in at least two of the *P. nodosus* ‘populations’ in Singapore, which demonstrate their ability to quickly recover from disturbances. In December 2006 and January 2007, an unusually heavy rainfall event over southern Johor in Peninsular Malaysia caused an excessive discharge of freshwater and hence sudden drop in salinity into the area around Pulau Sekudu [87]. Mass mortalities of intertidal organisms in the area were observed, including *P. nodosus*, but the populations appear to have restored their genetic diversity levels within 6 years of the devastation. At Cyrene reefs, only few individuals of *P. nodosus* were encountered in 2007 (K.P.P.T. 2007, personal observation), but in 2013 while sampling for this study, they were extremely abundant and genetically diverse (estimated population approx. 900 individuals [81]). The reason for this rapid population expansion at Cyrene reefs is, however, still unclear. Within Singapore waters, the most important population of *P. nodosus* is arguably at Cyrene reefs and it should be the main conservation target if resources are limited and prioritization of sites is required. The high genetic variability in such populations should be maintained because it will allow for adaptive changes to environmental change (reviewed in [88]).

Our study of genome-wide SNPs supports previous research using fewer genetic markers, showing patterns of high genetic diversity and likely high connectivity consistent among several broadcast-spawning marine organisms in Singapore, and supports the concept of unified conservation strategies for marine biodiversity. With the advent of next-generation sequencing technology, more population studies can be performed rapidly using genome-wide SNPs for non-model organisms (e.g. [20–22,59,61]), bypassing the time- and resource-consuming process of developing and using species-specific markers (e.g. [89,90]). The ddRADseq SNP loci obtained and used in this study can also be easily adapted elsewhere for comparison. Future work, especially on organisms that show similarly shallow genetic structure, can be complemented with hydrodynamic models of larval dispersal patterns, which are integrated with in-depth behavioural and life cycle studies, in order to obtain a more comprehensive understanding of population dynamics (e.g. [91]).

## Supplementary Material

Supplementary Materials

## Data accessibility

The data are available as the electronic supplementary material in the Dryad Digital Repository: http://dx.doi.org/10.5061/dryad.hr431.
