# Supplementary material for: Beyond the Coral Triangle: high genetic diversity and near panmixia in Singapore's populations of the broadcast spawning sea star Protoreaster nodosus
Source: R Soc Open Sci. 2016 Aug 17;3(8):160253. doi: 10.1098/rsos.160253 (PMC5108950; doi:10.1098/rsos.160253)
Supplement: Supplementary Materials [file rsos160253supp1.pdf]

Supplementary Materials

Table S1. Population diversity statistics based on COI data as calculated in DnaSP for all samples collected per population (Total), and the average of 30 random subsets of 15 individuals per population (Subset (15)). N = number of individuals, N_H = number of haplotypes with number of private haplotypes in square brackets, h = haplotype diversity, π = nucleotide diversity.

			N_H		h ± SD		
	**Sampling locality**	**N**	**Total**	**Subset (15)**	**Total**	**Subset (15)**	**π ± SD**
**1**	Beting Bronok*	1	1		-		-
**2**	Chek Jawa*	4	2		-		-
**3**	Pulau Sekudu	21	9 [5]	7.37 ± 0.85	0.80 ± 0.08	0.798 ± 0.053	0.003 ± 0.0007
**4**	Cyrene reefs	30	14 [10]	8.37 ± 1.27	0.86 ± 0.05	0.869 ± 0.045	0.003 ± 0.0005
**5**	Pulau Semakau	22	6 [1]	5.3 ± 0.6	0.80 ± 0.05	0.799 ± 0.028	0.002 ± 0.0005
**6**	Karimunjawa	35	15	7.47 ± 1.61	0.74 ± 0.08	0.715 ± 0.114	0.002 ± 0.0004
**7**	Nusa Tengerrah	23	8	5.67 ± 1.15	0.74 ± 0.08	0.629 ± 0.151	0.002 ± 0.0004
**8**	Sabolo Kecil, Flores*	9	3		-		-
**9**	South Sulawesi	30	13	8.37 ± 1.25	0.83 ± 0.06	0.847 ± 0.052	0.003 ± 0.0007
**10**	Manado	28	10	6.5 ± 1.48	0.70 ± 0.09	0.703 ± 0.118	0.002 ± 0.0004
**11**	Halmahera	64	27	8.23 ± 1.7	0.77 ± 0.06	0.765 ± 0.107	0.002 ± 0.0002
**12**	Raja Ampat	54	13	4.67 ± 1.37	0.45 ± 0.09	0.462 ± 0.152	0.001 ± 0.0003
**13**	Teluk Cenderawasih*	7	4		-		-
**14**	Numfor^	15	6	6	0.79 ± 0.08	0.79	0.003 ± 0.0006
**15**	Biak	19	9	7.67 ± 0.96	0.77 ± 0.09	0.769 ± 0.056	0.003 ± 0.0005
**16**	Yapen	22	10	7.7 ± 1.18	0.86 ± 0.05	0.849 ± 0.043	0.003 ± 0.0003
	Overall	384	91		0.77 ± 0.02		0.003 ± 0.0001

*Samples not included in population statistics calculations

^Numfor used as base of 15 samples for random subsampling in the other populations

Table S2. Barcodes and corresponding Illumina index sequences used for ddRADseq. Highlighted in grey are the three samples that were excluded from population genetic analyses due to low coverage.

	Barcode	Illumina Index	
SE01	CGAAT	6	GCCAAT
SE05	CTGAT	7	CAGATC
SE07	GAGAT	1	ATCACG
SE08	CTTGG	1	ATCACG
SE09	GGATA	7	CAGATC
SE10	GCTGA	1	ATCACG
SE14	GTCGA	7	CAGATC
SE15	GTAGT	1	ATCACG
SE17	TACGT	7	CAGATC
SE18	TCACG	7	CAGATC
SE19	GACAC	5	ACAGTG
SE20	GCCGT	5	ACAGTG
SE21	TCTGC	7	CAGATC
PS12	ACTTC	1	ATCACG
PS14	AGCTA	1	ATCACG
PS16	ATTAC	5	ACAGTG
PS17	ACACA	1	ATCACG
PS18	AATTA	6	GCCAAT
PS19	CATAT	5	ACAGTG
PS21	ATGAG	1	ATCACG
PS25	CGTAC	1	ATCACG
PS26	ACGGT	6	GCCAAT
PS28	CGGCT	5	ACAGTG
PS29	CTGTC	1	ATCACG
CY26	ACTGG	5	ACAGTG
CY27	ATACG	5	ACAGTG
TP07	TAGTA	5	ACAGTG
TP08	CGGTA	6	GCCAAT
TP09	CGTCG	6	GCCAAT
TP10	GAGTC	6	GCCAAT
TP11	GTCCG	1	ATCACG
TP12	GGCCA	6	GCCAAT
TP13	TACCG	6	GCCAAT
TP14	TCCGG	1	ATCACG
TP15	TATAC	6	GCCAAT
TP16	TTACC	6	GCCAAT

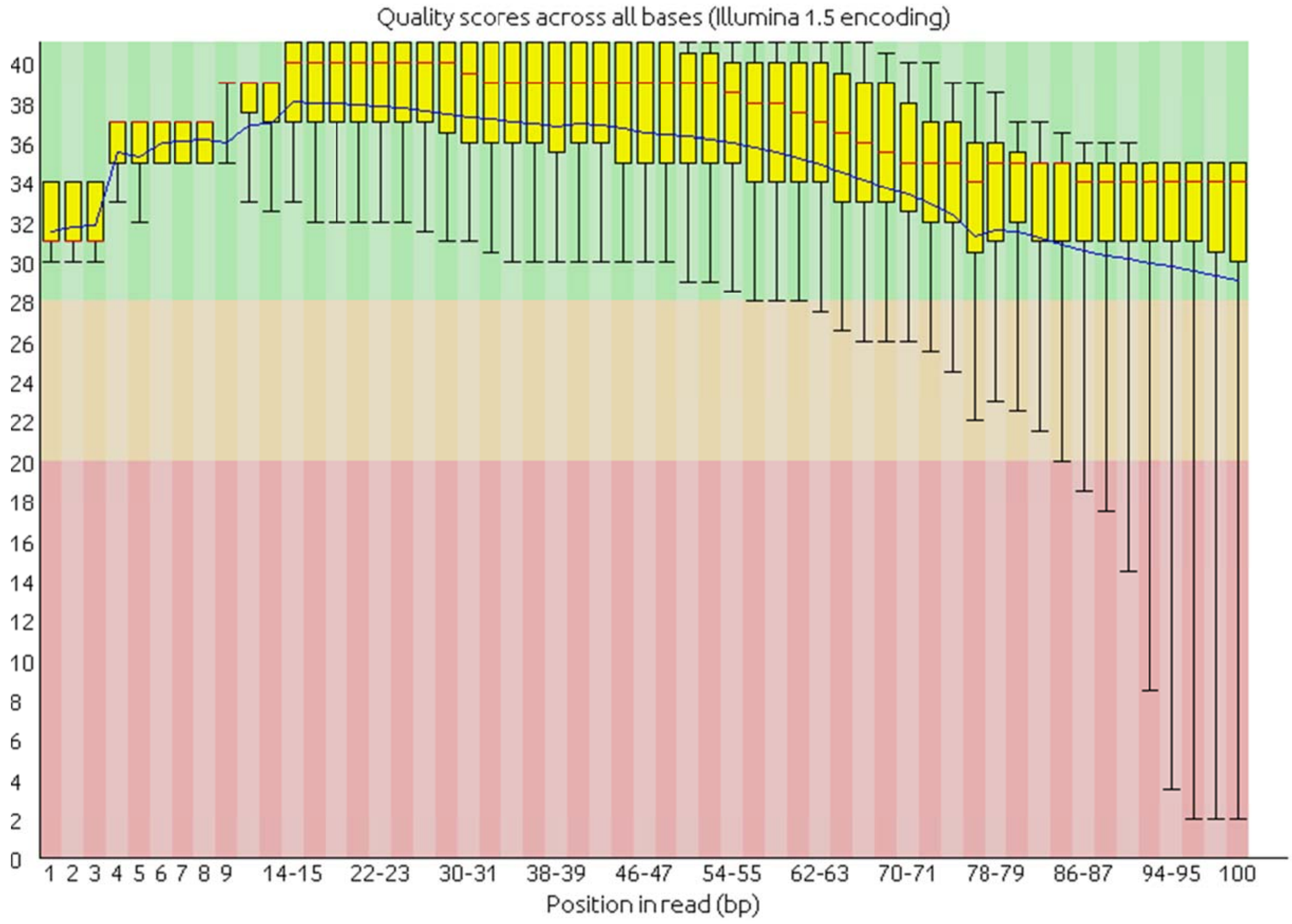

Fig. S1 Quality scores for Read 1 of the HiSeq run, as summarised by FastQC

2a)

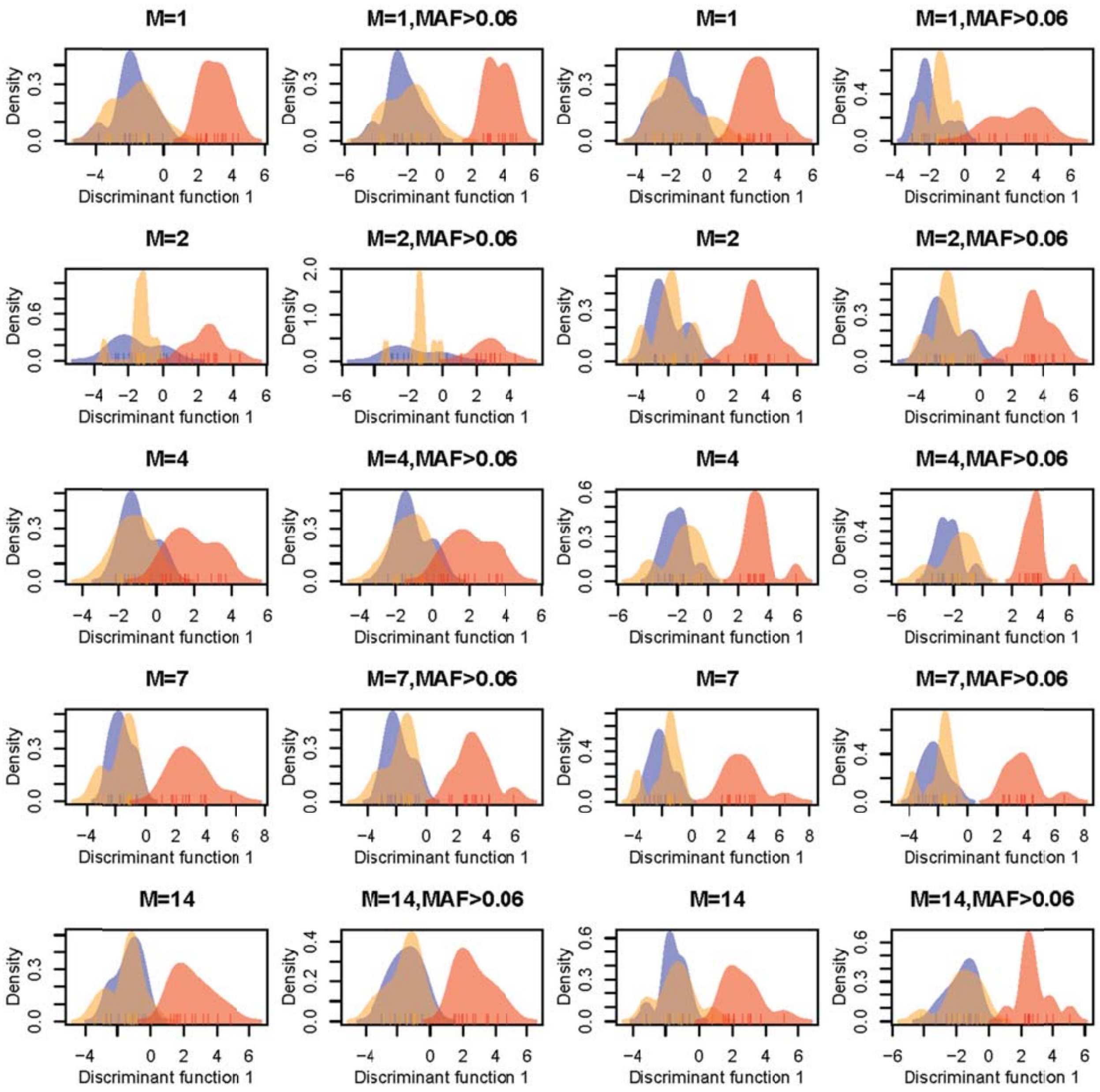

M=1
M=1,MAF>0.06
M=2
M=2,MAF>0.06
M=4
M=4,MAF>0.06
M=7
M=7,MAF>0.06
M=14
M=14,MAF>0.06
Density
Discriminant function 1

2b)

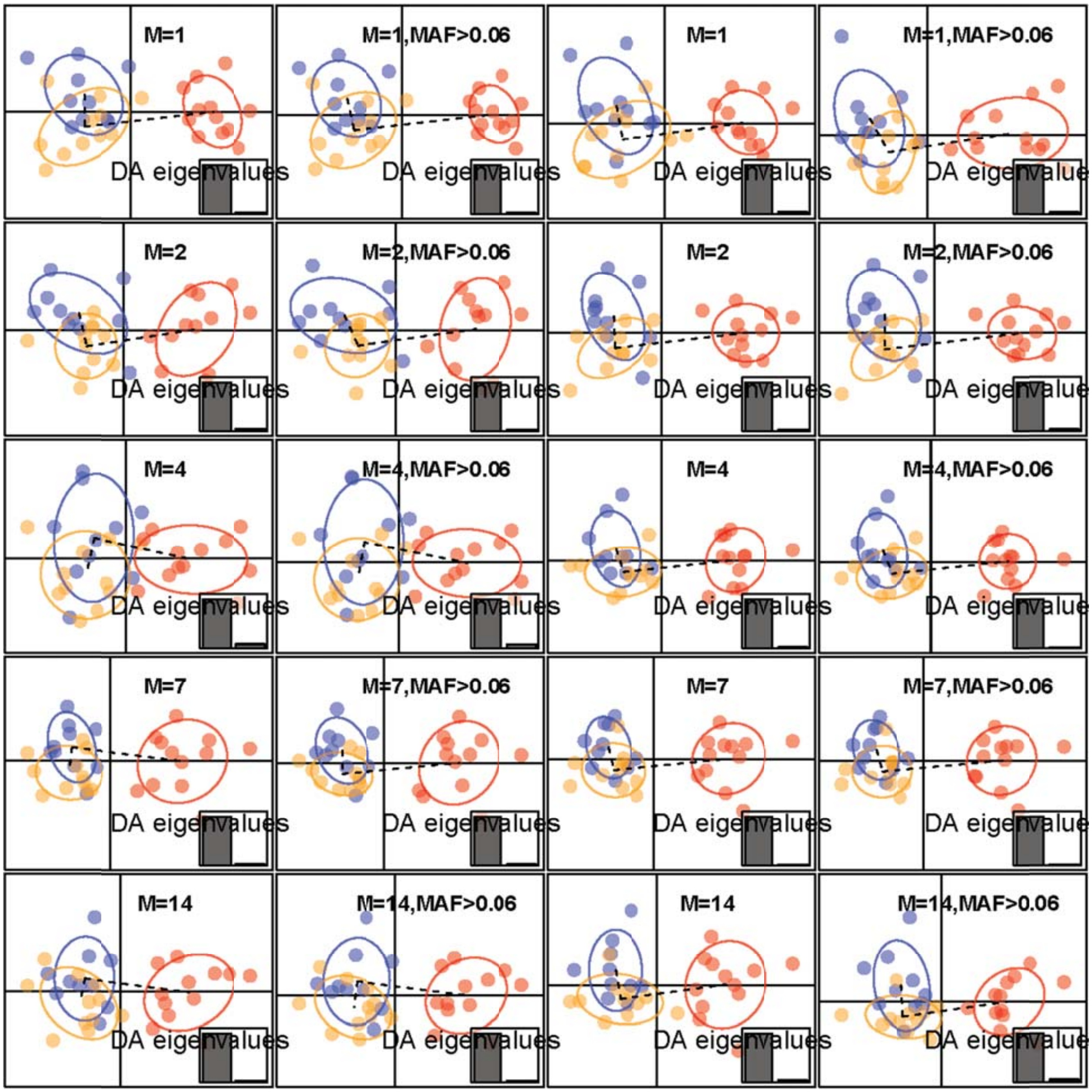

Fig. S2 Mismatch threshold sensitivity analysis assessed via DAPC plots when (a) one and (b) two discriminant functions were retained. Samples are colour-coded as red = Pulau Sekudu, blue = Cyrene reefs, yellow = Pulau Semakau. In rows – different mismatch thresholds (M=1, 2, 4, 7, 14), columns 1 and 2 – loci obtained without filtering for potential chimeras, columns 3 and 4 – loci obtained after filtering for potential chimeras; columns 1 and 3 – without application of minor allele frequency, columns 2 and 4 – minor allele frequency filter >0.06 applied

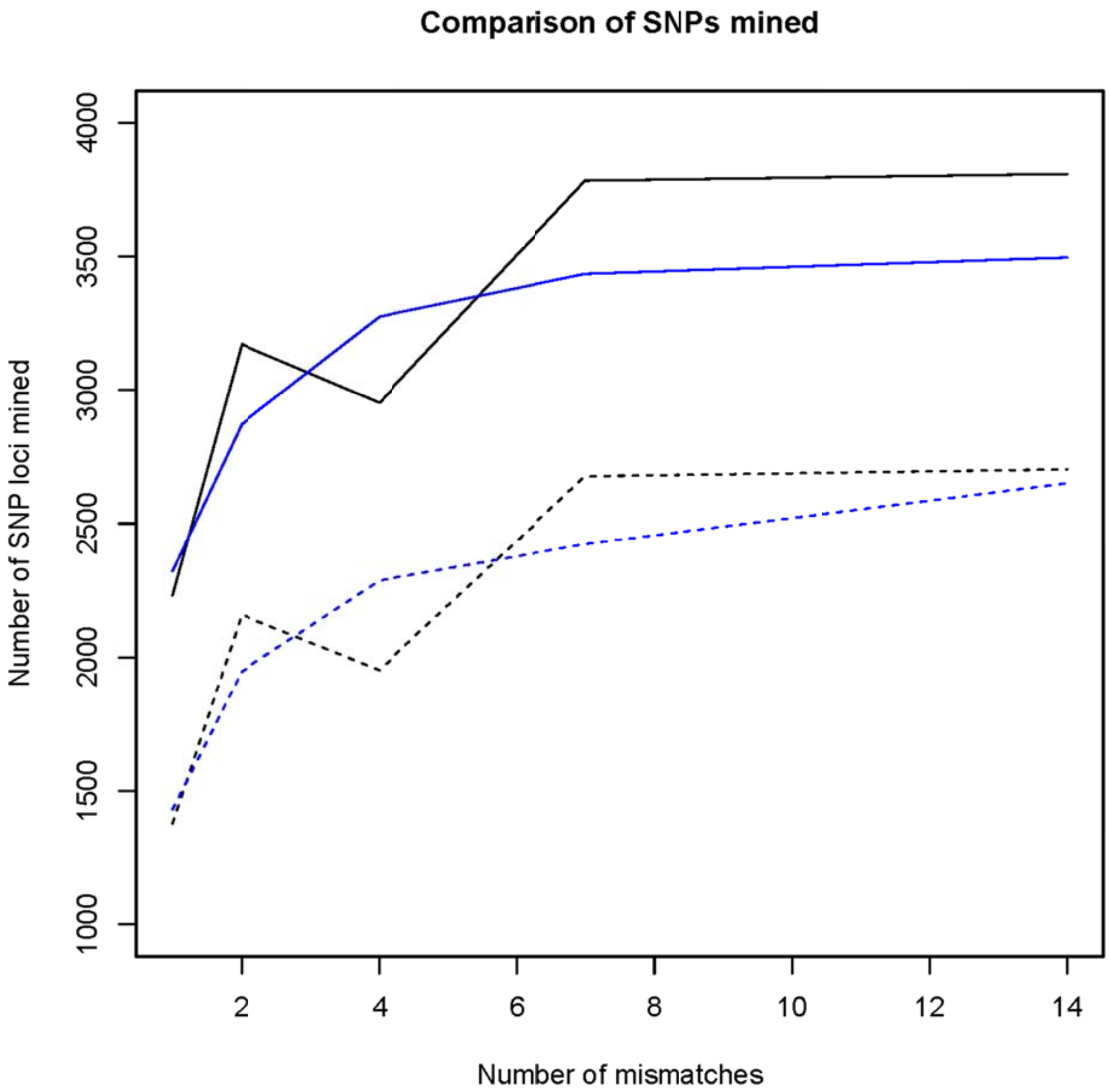

Fig. S3 Number of SNP loci mined across different SNP calling parameters. Mismatch thresholds are represented on the x-axis, blue lines represent data sets in which potential chimeras were removed, while dotted lines represent data sets in which a minor allele frequency of >0.06 was applied

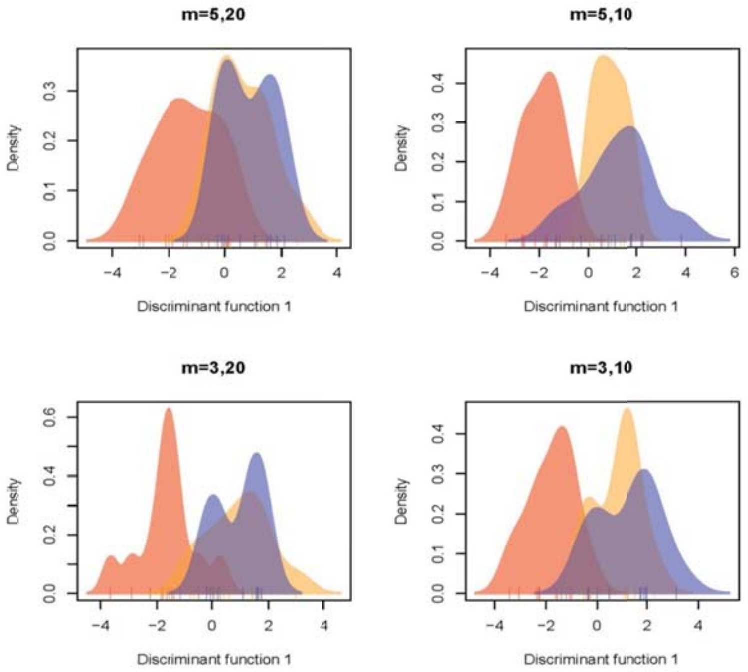

a)

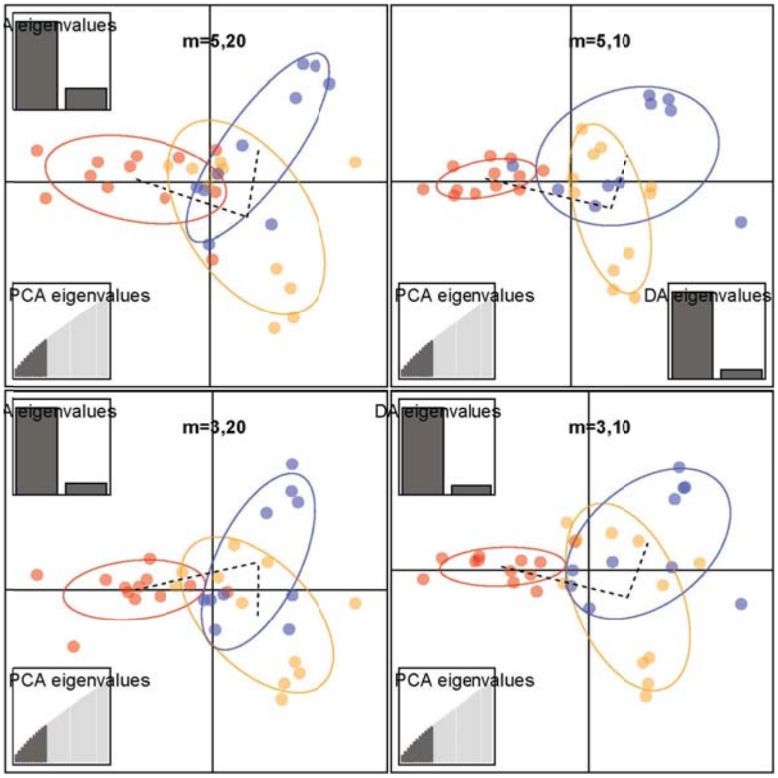

b)

Fig. S4 Effects of minimum stack depths as assessed via DAPC plots when (a) one and (b) two discriminant functions were retained. Samples are colour-coded as red = Pulau Sekudu, blue = Cyrene reefs, yellow = Pulau Semakau

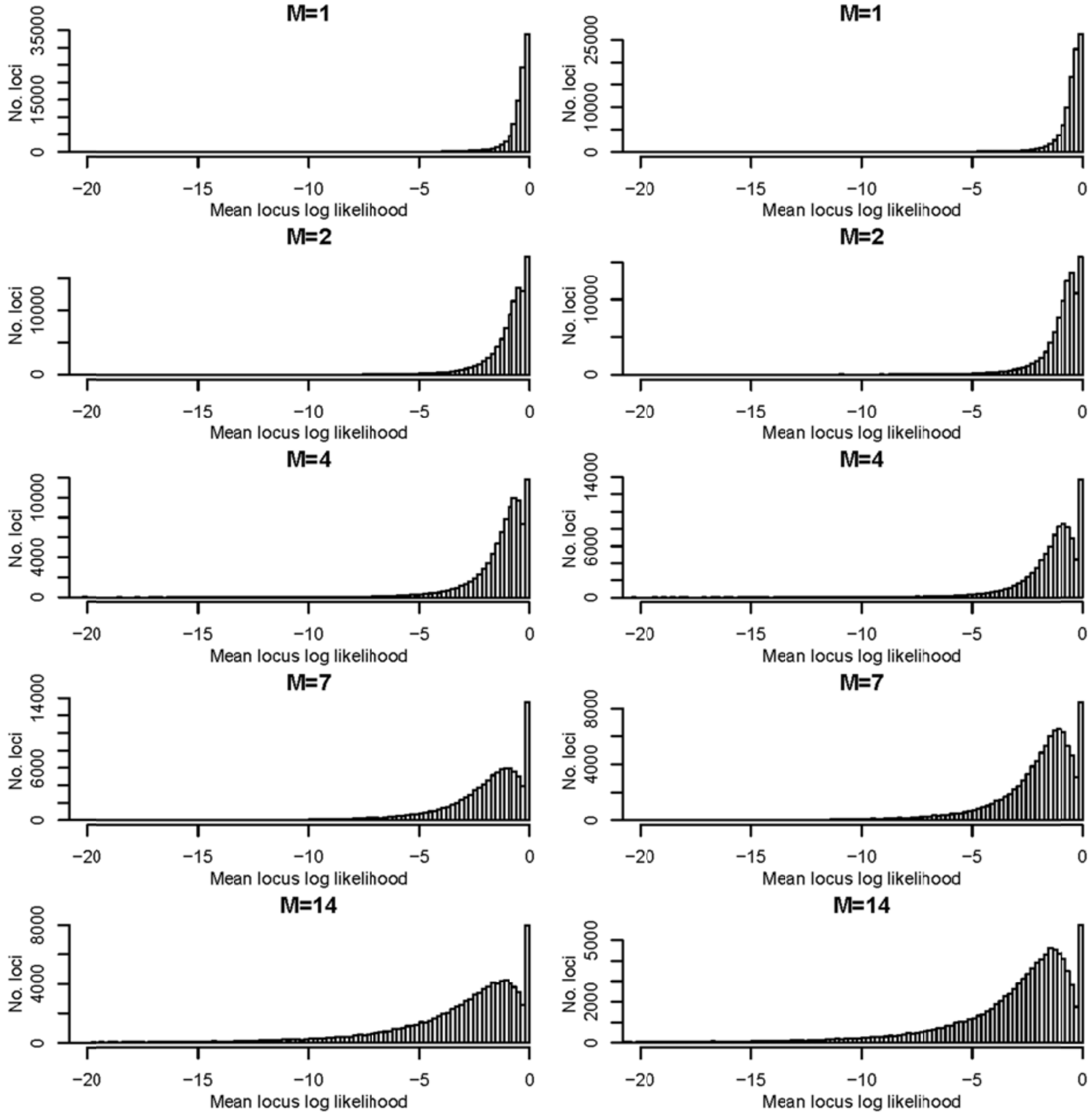

Fig. S5 Log likelihood distribution profiles for loci from all individuals under different mismatch thresholds in STACKS (in rows, M = 1, 2, 4, 7, 14). Left column – loci obtained without filtering for potential chimeras; Right column – loci obtained after filtering for potential chimeras

6a) Cyrene reefs

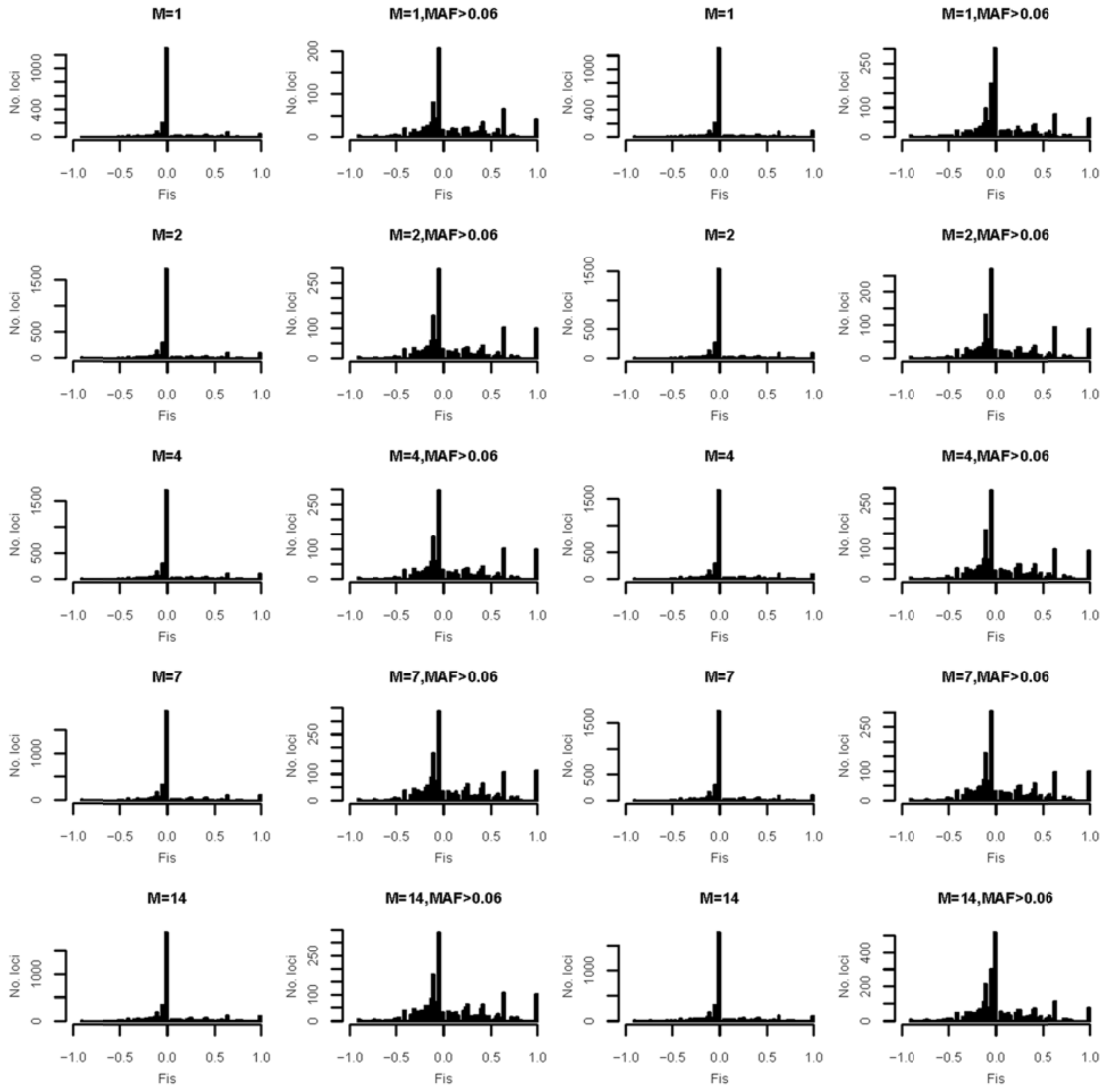

6b) Pulau Semakau

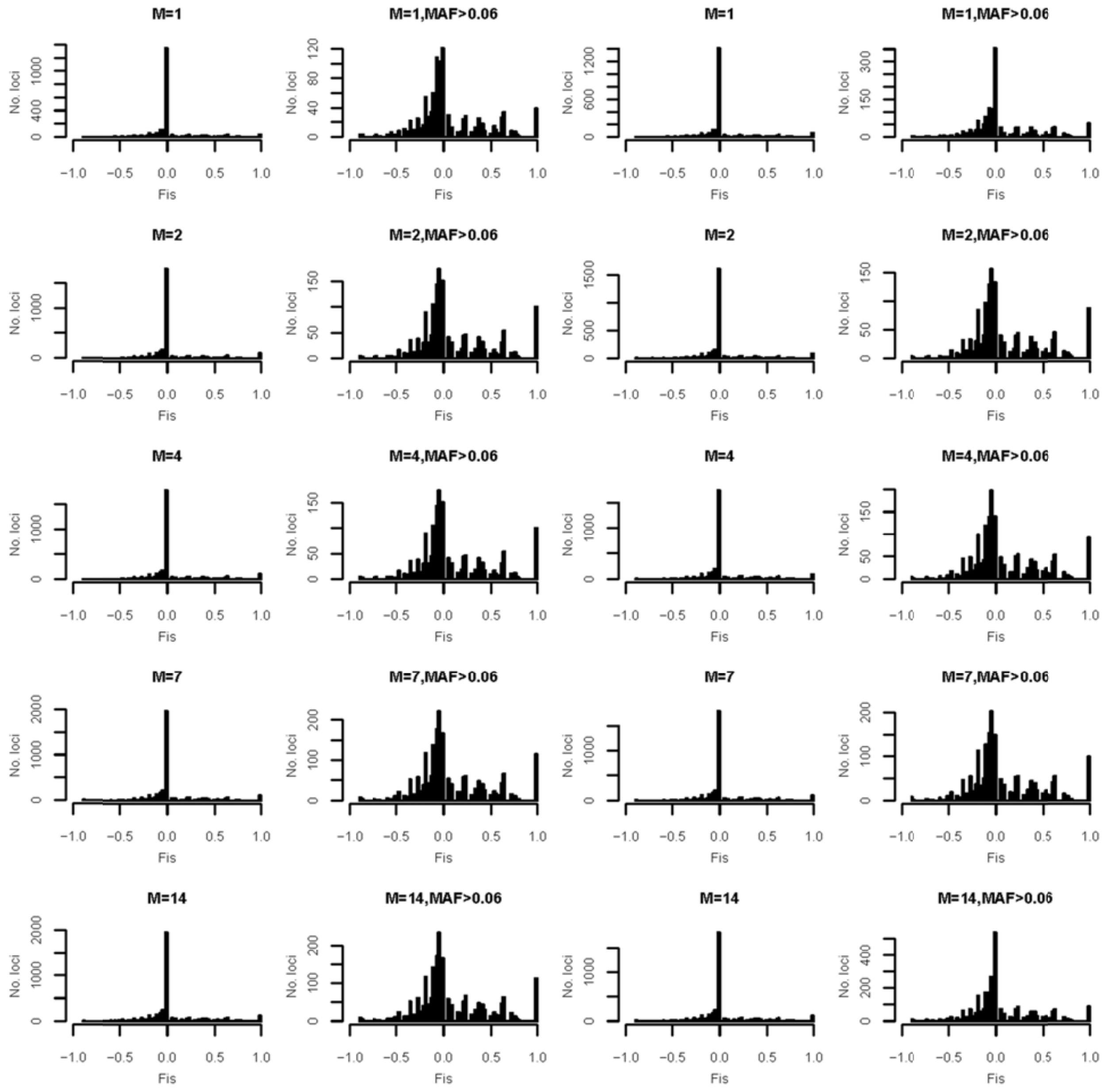

6c) Pulau Sekudu

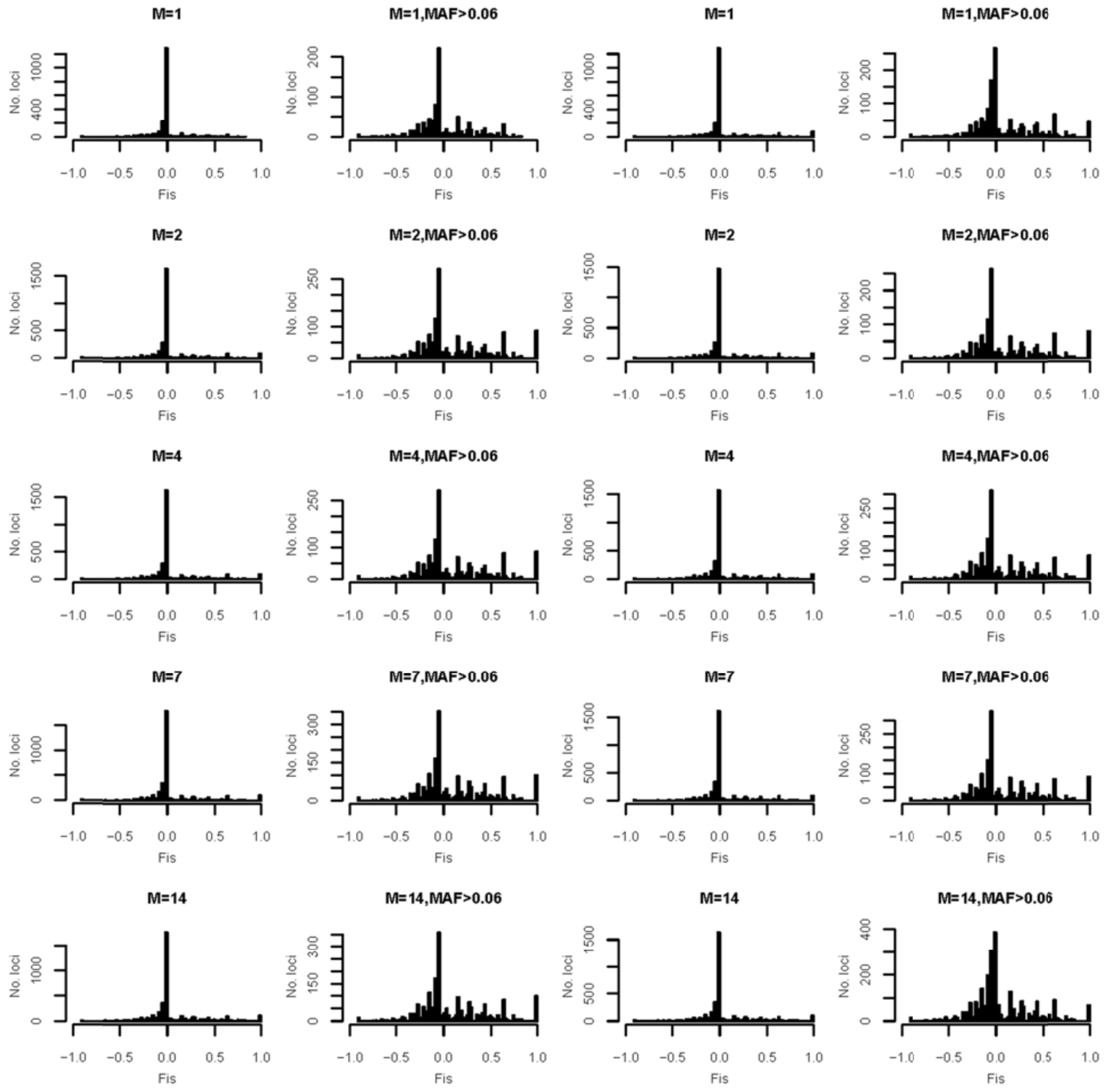

Fig. S6 F_{IS} distribution profiles at each of the sites (a) Cyrene Reefs, (b) Pulau Semakau, (c) Pulau Sekudu. In rows – different mismatch thresholds (M=1, 2, 4, 7, 14), columns 1 and 2 – loci obtained without filtering for potential chimeras, columns 3 and 4 – loci obtained after filtering for potential chimeras; columns 1 and 3 – without application of minor allele frequency, columns 2 and 4 – minor allele frequency filter >0.06 applied

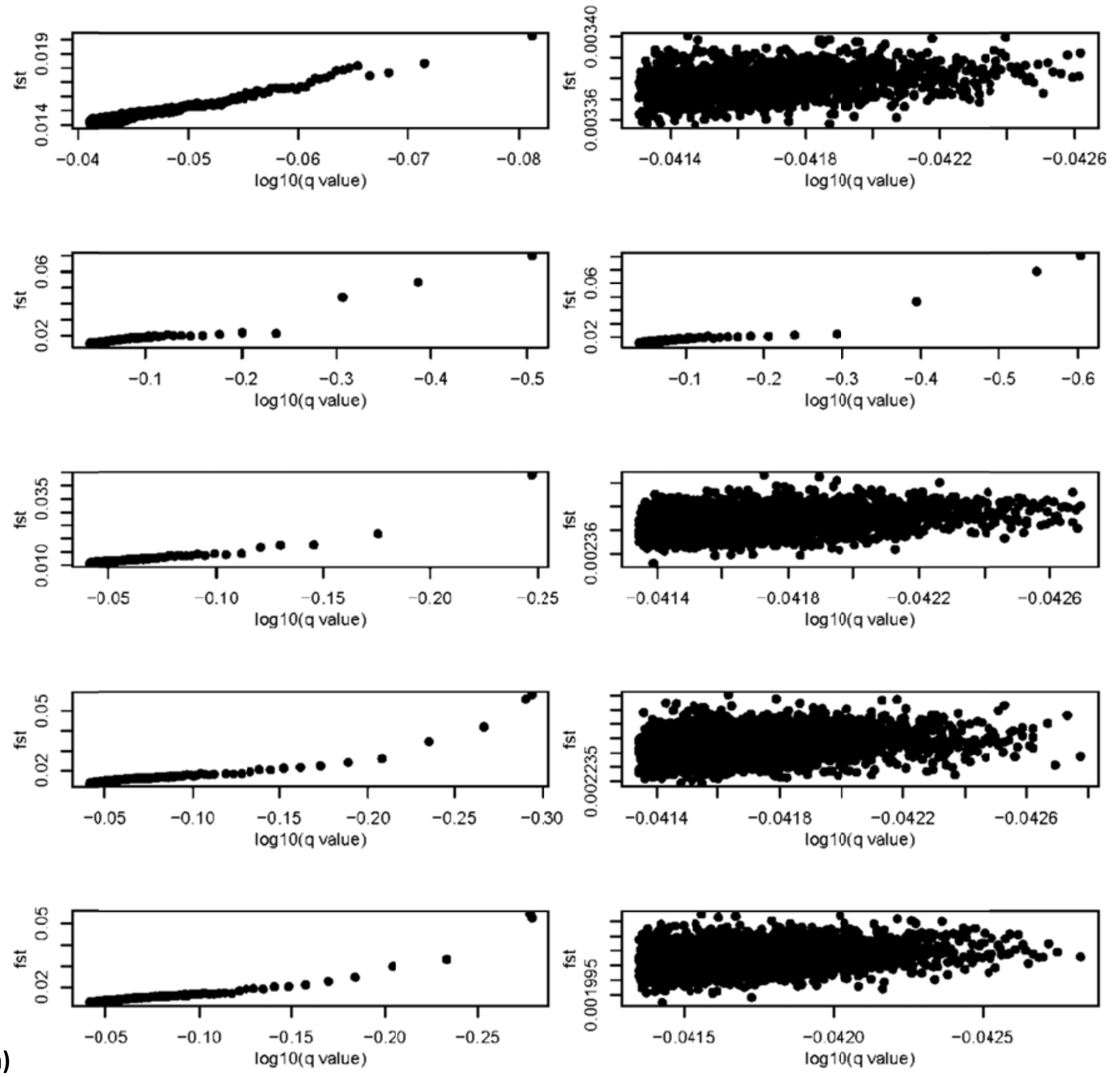

a)

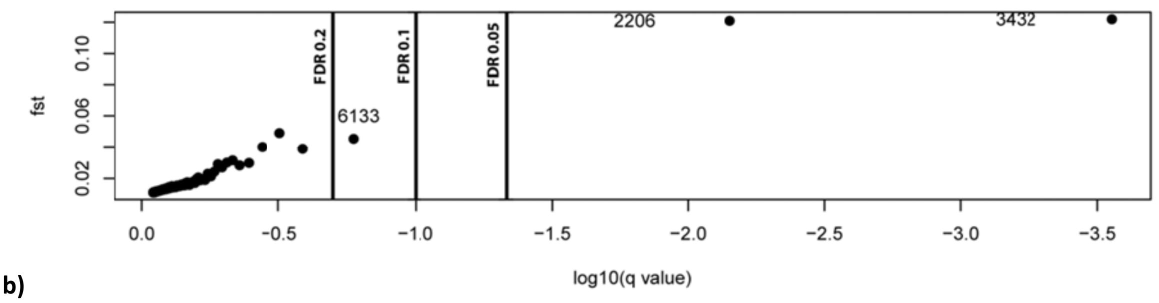

b)

Fig. S7 Bayescan analyses of SNP loci from the (a) data sets including (left column) and excluding (right column) reads with potential chimera sites, at mismatch thresholds M=1, 2, 4, 7, and 14 (rows 1 to 5). Even at 20% FDR cut-off, no outliers were observed, and from the (b) main data set analysed

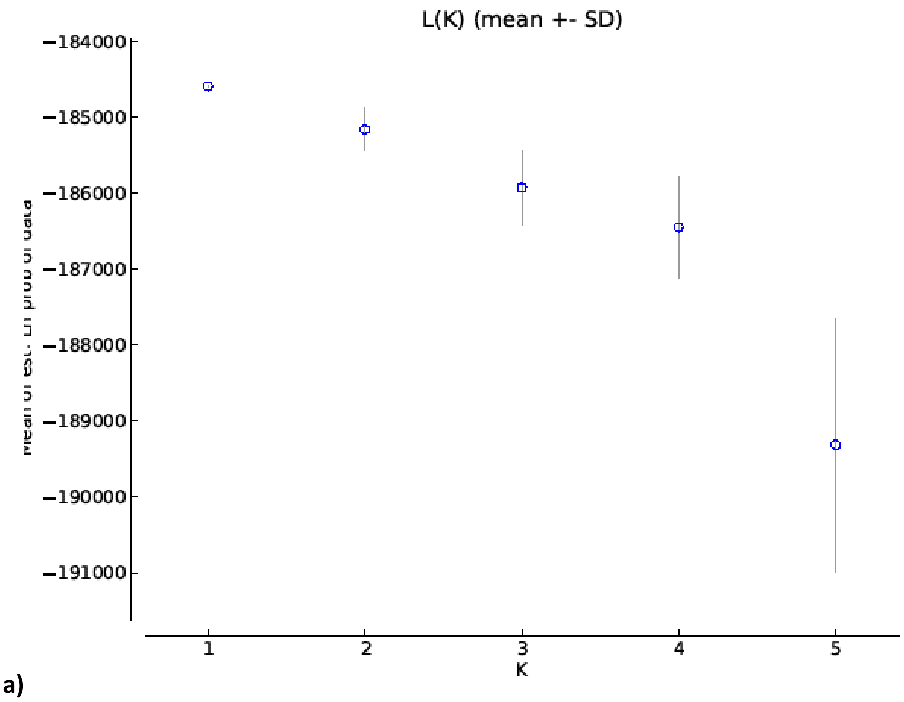

a)

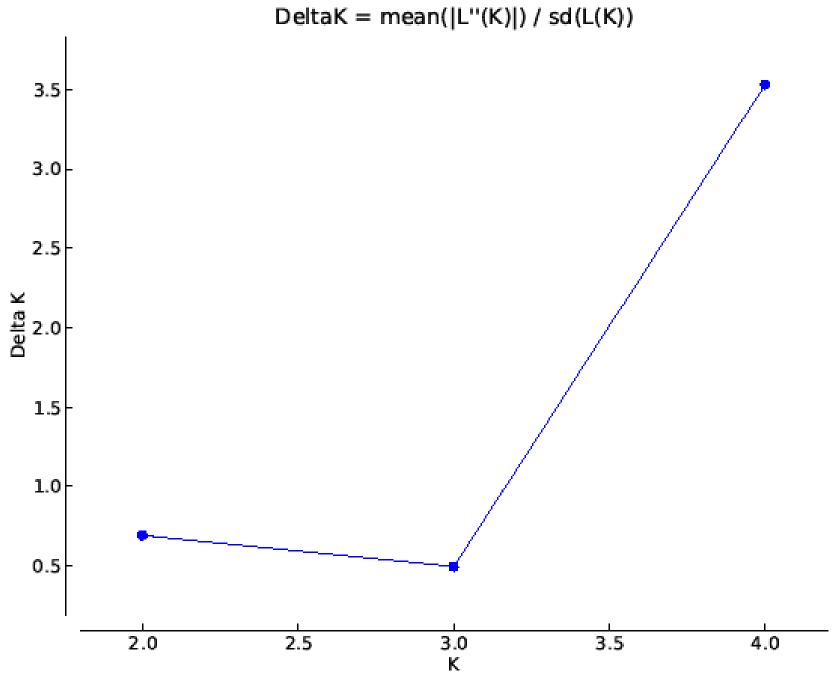

b)

Fig. S8 STRUCTURE HARVESTER analyses based on 6140 ddRADseq SNP loci harvested under the mismatch threshold M=4 and MAF>0.06, for K=1–5 over ten iterations. (a) Plot of mean likelihood L(K) and variance per K, (b) plot of the change in the probability of K that estimates K=4 as optimal number of genetic clusters

SNP calls were also made using pyRAD at several setting combinations to assess the effects of including indels in the clustering of SNP loci. Settings tested include minimum coverages per cluster of 10 and 2, clustering thresholds (similarity) of 0.9 and 0.94, and major base call depths of 10 and 5. Between 4908 to 11250 SNP loci were called using these parameter settings. Since all DAPC plots gave similar results, here we present the SNP analyses called with minimum cluster coverage of 10.

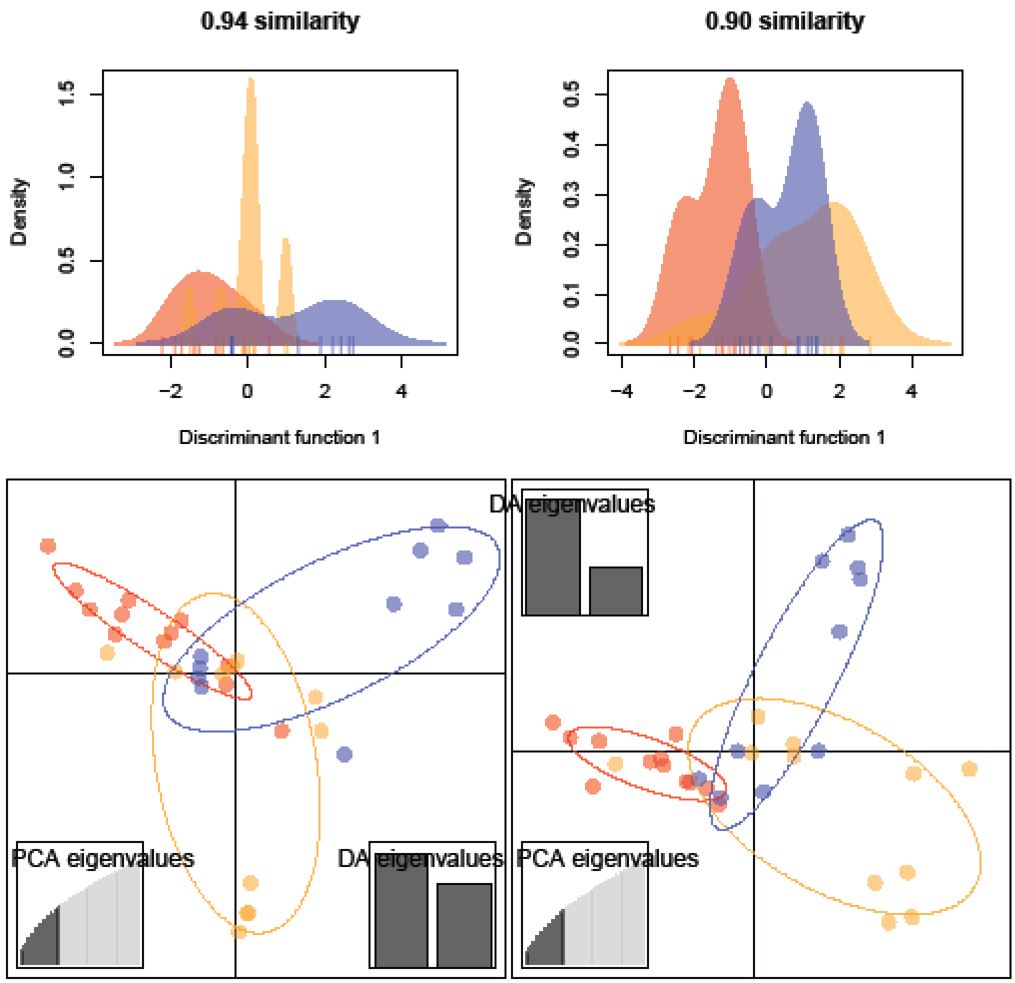

Fig. S9 pyRAD SNP calls as assessed via DAPC plots for when minimum cluster coverage=10, when (top) one and (bottom) two discriminant functions were retained. Samples are colour-coded as red = Pulau Sekudu, blue = Cyrene reefs, yellow = Pulau Semakau
